# Is the excellent air quality a protective factor of health problems for Taitung County in eastern Taiwan? Perspectives from visual analytics

**DOI:** 10.1016/j.heliyon.2023.e13866

**Published:** 2023-02-18

**Authors:** Ching-Hsiang Yu, Shun-Chuan Chang, En-Chih Liao

**Affiliations:** a Department of Emergency Medicine, MacKay Memorial Hospital, Taipei, Taiwan; b Holistic Education Center, MacKay Medical College, New Taipei City, Taiwan; c Department of Medicine, MacKay Medical College, New Taipei City, Taiwan; d Institute of Biomedical Sciences, MacKay Medical College, New Taipei City, Taiwan

**Keywords:** Taitung, Air quality index (AQI), Air pollution, Asthma, Happiness index, Income, Generalized association plots (GAP), Rural-urban disparity

## Abstract

Taitung, an agricultural country in Eastern Taiwan, was famous for its fresh air with less industrial and petrochemical pollution. Air pollution may induce cardiovascular disease, chronic obstructive pulmonary disease (COPD), asthma, and stroke, poor air quality also resulted in a higher depression rate and less feeling of happiness; therefore, our study aims to use visualization tools to demonstrate the association between air quality index (AQI) and the among negative factors and try to find that whether Taitung got the benefit of good air quality on health issues. We retrieved data from the government of Taiwan and other open sources in the year 2019, then visual maps and generalized association plots with clusters demonstrated the relationship between each factor and each county/city. Taitung had the lowest AQI and asthma attack rate, but AQI had a negative relationship to air pollution-caused death (R = −0.379), happiness index (R = −0.358), and income (R = −0.251). The GAP analysis revealed that smoke and overweight were the nearest to air pollution causing death, also counties and cities were divided into two major clusters initially based on the air pollution-related variables. In conclusion, the World Health Organization (WHO) definition and the weight of each air pollution cause death may not be suitable for Taiwan due to too many confounding factors.

## 1 Introduction

Air quality index (AQI) is an indicator of air pollution, according to the Environmental Protection Administration (EPA) of Taiwan, the concentration of ozone (O3), suspended fine particles of Particulate Matter≦2.5 μm (PM_2.5_), suspended particles of PM_10_, carbon monoxide (CO), sulfur dioxide (SO_2_) and nitrogen dioxide (NO_2_) in the same day will be converted into many sub-indicators based on their impact on the human body, then the maximum of sub-indicator will become the AQI of that day [1].

Taitung County is located in the southeast of Taiwan, its industrial structure is mainly based on the farming industry, which resulted in less industrial air pollution and better air quality, thus these natural advantages of the environment were also featured for the promotion of the tourism industry in recent years [2]. As to the assessment of air quality by the residents, Taitung and Hualien (another rural county in eastern Taiwan) Counties belong to the "excellent" class [3], the data from Environmental Protection Administration also revealed that Taitung County had the lowest AQI in Taiwan, which caused much better visibility than the highly urbanized center (Taipei City, in northern Taiwan) and highly industrialized center (Kaohsiung City, in southwestern Taiwan) [4].

Much evidence supported that air pollution had multiple adverse effects on the human body, World Health Organization (WHO) pointed out that air pollution was estimated to cause about 29% of lung cancer deaths, 43% of chronic obstructive pulmonary disease (COPD) deaths, about 25% of ischemic heart disease deaths and 24% of stroke deaths [5]. Air pollution may exacerbate and even develop asthma, UK's Committee on the Medical Effects of Air Pollutants proposed four main mechanisms: oxidative stress and damage, airway remodeling, inflammatory pathways, and immunological responses, and enhancement of respiratory sensitization to aeroallergens [6,7]. Research in Taiwan also showed the same finding, a study based on the National Health Insurance database revealed that high levels of NO_X_ (NO and NO_2_) resulted in significant odds ratios of asthma exacerbation up to 1.45 in preschool children [8], another mass screening study among all middle school students demonstrated that students who lived in heavy air pollution areas were 1.8 times more likely to have a history of asthma than who lived in an area without pollution [9].

Happiness can be influenced by air pollution, when the concentration of NOX increased by 1 μg/m^3^, Chinese residents thought that their possibility of happiness would decrease by 0.034% [10], and a Spanish study found that people would like to pay 1.4% of their income for reducing 1% of air pollution [11]. Moreover, air pollutants and ambient air pollution levels were positively linked to suicide mortality and risk which was supported by studies in Japan, China, and Belgium [[12], [13], [14]].

Therefore, our study aims to evaluate whether good air quality provided health benefits to Taitung people or not and investigate the association between AQI and some bio-psycho-social indicators.

## 2 Methods

### 2.1 Data collection

The data of AQI and PM_2.5_ were downloaded from Taiwan Air Quality Monitoring Network [1], which was preserved by the Environmental Protection Administration, Executive Yuan. We used the average AQI and PM_2.5_ of each county or city in 2019 for the study.

The data on causes of death were obtained from the Department of Statistics, Ministry of Health and Welfare [15]. The standardized mortality ratio of lung cancer deaths, COPD deaths, ischemic heart disease deaths, and stroke deaths of each county or city was multiplied by the weights of each disease (29%, 43%, 25%, 24%) based on WHO online database "Burden of disease from joint household and ambient Air Pollution” and summed together.

The data on asthma attack incidence rate was retrieved from the Quality Performance of National Health Insurance Disclosure Website. It was a rate of patients involved in the Improvement Plan of Medical Benefit of National Health Insurance (NHI) for Asthma suffering from asthma attacks then being sent to the emergency department [16]. The rates of the four seasons in 2019 were averaged and the means were used for further analysis.

The data on the happiness index of 2019 was gotten from Economy Daily News (Taiwan), this index is based on the structure of the Organization for Economic Co-operation and Development (OECD) Your Better Life Index [17], consisted of two major parts, objective well-being, and subjective well-being, the first one was calculated by governmental statistics, the other was analyzed by public opinion poll [18].

The demographic data including income, smoking rate, and prevalence of overweight was accessed from the Department of Statistics, Ministry of Health and Welfare (Department of Statistics). The percentage of residents who received higher education was downloaded from the Department of Household Registration, Ministry of the Interior [19].

Excel Office 365 (Microsoft, Redmond, WA, USA) with a map template was administered for drawing the visual map of each indicator, the green-yellow-red spectrum was used for filling in color with its corresponding value [20]. Another online map generator (https://pixelmap.amcharts.com/#) was applied for the demonstration of the overview of the 20 counties/cities and their grouping.

### 2.2 Statistical analysis

SPSS version 24.0 (IBM, Armonk, NY, USA) was used for calculating the Pearson’s correlation coefficient between AQI and each indicator, then we should use the absolute criterion for correlations as the following [21]:

0–0.19: no correlation,

0.2–0.39: low correlation, 0.40–0.59: moderate correlation,

0.60–0.79: moderately high, ≥0.80: high correlation.

The student’s t-test was chosen for p-value under the statistical guidelines [22,23], α level of 0.05 was set for all statistical tests [24], and all indicators were standardized for generalized association plots.

### 2.3 Generalized association plots (GAP)

Generalized association plots (GAP) was a Java-designed software developed by Chun-Houh Chen from the Institute of Statistical Science Academia Sinica [25], white-black spectrum was used in the raw data matrix, blue-white-red bidirectional color spectrum was chosen for displaying the correlation coefficient in proximity matrices for columns (9 health related-factors) and rows (20 cities and counties), then the average-linkage method was used for hierarchical clustering.

## 3 Results

### 3.1 Subsection

**Fig. 1:**
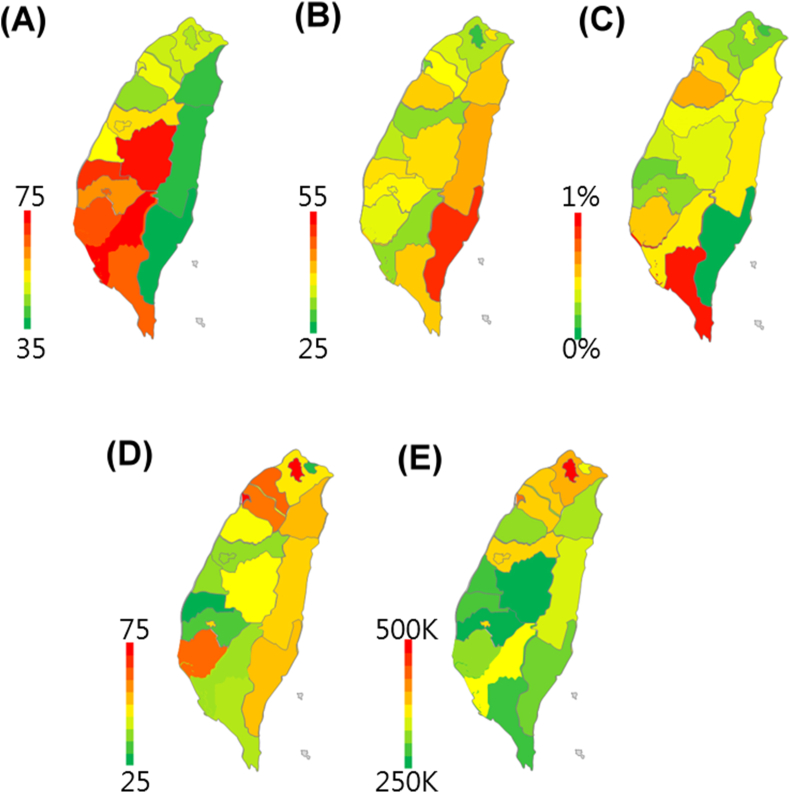
The visual maps of (A) air pollution, (B) air pollution caused death, (C) asthma attack, (D) happiness, and (E) income. AQI ranged from 35 to 75, the SMR of air pollution caused death per million people ranged from 25 to 55, the asthma attack rate ranged from 0% to 1%, the happiness index ranged from 25 to 75, and the annual income per person ranged from 250 thousand to 550 thousand of New Taiwan Dollar (30 NTD ≒ 1 USD). The (A), (B), and (C) were negative indicators. The redder was the worse and the greener was the better. The (D) and (E) were positive indicators, so the redder became the better.

In Fig. 1A, the greener area had the better air quality, so the air quality of Yilan County, Hualien County, and Taitung County (from the northeastern to southeastern coast area) was good, and the region with the poorest air quality was the southwestern area, especially Kaohsiung City.

The redder area in Fig. 1B implied a higher standardized mortality ratio of air pollution caused death, and Taitung had the highest value, also the other two counties on the eastern coast (Yilan County and Hualien County) were in yellow to red. On the contrary, Kaohsiung City which was in red on the AQI map was filled with light green in the visual map of air pollution caused death, the statistical result also revealed that AQI and air pollution caused death had a low negative correlation (R = −0.379).

Fig. 1C is the visual map of the asthma attack rate, which showed that Taitung County was in dark green, and Pingtung County was in dark red, then AQI and asthma attack had a low positive correlation (R = 0.263). The visual map of the happiness index was demonstrated in Fig. 1D, the yellow color in the three east coast counties corresponded to the moderate level of the happiness index, and Taipei City was the most well-being area which was in dark red. AQI and happiness index had a low negative correlation (R = −0.358).

We found that the richest counties/cities were in northern Taiwan which were in orange to red, and the three east coast counties had the lower income, so they were in the green (Fig. 1E). AQI and income had a low negative correlation (R = −0.251).

Although there was no significant correlation between AQI and the above four indicators, interestingly, happiness index and income had a moderate positive correlation (R = 0.595).

### 3.2 The association of air health-related factors

**Fig. 2:**
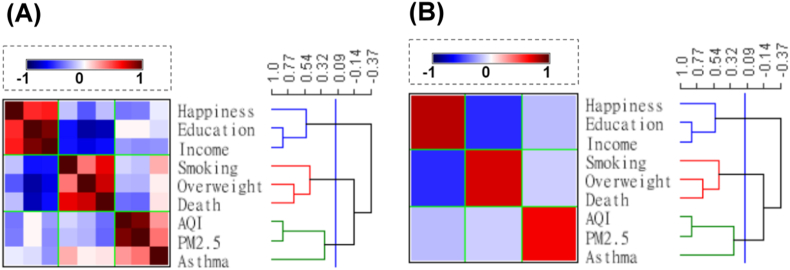
The proximity matrix of health-related indicators. When the cut point was set as a correlation coefficient (R) around 0.1, the 9 indicators could be divided into 3 clusters. (A). The raw Indicator × Indicator matrix; (B). The proximity matrix after partition by cut point and sufficient with the means of each square. Happiness = Happiness index, Education = percentage of people receiving a college education, Income = the annual income per person, Smoking = smoking rate, Overweight = overweight rate, Death = SMR of air pollution caused death, AQI = air quality index, Asthma = asthma attack rate.

Fig. 2A showed that three clusters were divided, cluster 1 was separated initially comprising happiness index, higher education, and income, cluster 2 was composed of smoking, overweight, and air pollution caused death, cluster 3 included AQI, PM_2.5_, and asthma attack rate. Based on their composition, cluster 1 was named “positive indicators”, cluster 2 was named “negative indicators”, and cluster 3 was named “air pollution indicators”.

To demonstrate the relationship between each cluster clearly, the matrix under-went partition and sufficient with the means of each square then became Fig. 2A. The color square between “positive indicators” and “negative indicators” was dark blue, indicating they had a moderately to highly positive association, and the color square between “positive indicators” and “air pollution indicators”, “negative indicators” and “air pollution indicators” were light blue, implying their associations were weak (Fig. 2B).

### 3.3 The association of air health-related factors

After hierarchical clustering, four groups were successfully divided as the following (Table 1):

**Table 1:** The characteristics of the four groups of counties/cities.

Group/Cluster	Positive indicators	Negative indicators	Air pollution-related indicators
Group 1	Low	High	High
Group 2	Moderate	High	Low
Group 3	Moderate to high	Low	High
Group 4	High	Low	Low to moderate

Group 1: Nantou County, Yunlin County, Changhua County, Pingtung County, Chiayi County.

Group 2: Keelung City, Penghu County, Miaoli County, Hualien County, Taitung County, Yilan County.

Group 3: Tainan City, Chiayi City, Kaohsiung City, Taichung City.

Group 4: Hsinchu County, Taoyuan City, Hsinchu City, Taipei City, New Taipei City.

According to the raw data matrix (Indicators × Counties/cities, in the white-black spectrum), Group 1 had a low level of “positive indicators”, a high level of “negative indicators” and “air pollution indicators”; Group 2 had a moderate level of “positive indicators”, high level of “negative indicators”, and low level of “air pollution indicators”; Group 3 had a moderate to the high level of “positive indicators”, low level of “negative indicators”, and high level of “air pollution indicators”; Group 4 had a high level of “positive indicators”, low level of “negative indicators”, and low to moderate level of “air pollution indicators”.

The proximity matrix of row (Indicators × Indicators, at the ride side in the blue-white-red spectrum) also underwent partition and sufficient as Fig. 4, Fig. 5, then the mildly negative correlation between Group 1 & 2, Group 2 & 4, also the highly negative correlation between Group 2 & 3 were demonstrated. Moreover, there was a mildly to the moderately positive correlation between Groups 1 & 3, and Groups 3 & 4.

### 3.4 Finding the potential confounding factors of air pollution-caused death

Because the correlation between AQI and air pollution caused death was negative, implying that there should be some confounding factors that disturbed the relationship. Four candidates including smoking, overweight, income, and higher education were chosen, the first two were risk factors and the last two seemed to be protective factors. Table 2 showed that both smoking and overweight had moderately to highly associations (R = 0.70 and 0.75) to air pollution caused death with significant p-value (0.001 and < 0.001), income and higher education had significantly moderately to highly associations (R = −0.64 and −0.80, p-value = 0.002 and < 0.001) in contrast.

**Table 2:** The associations of air pollution-caused death and potential confounding factors.

Potential confounding factors	Correlation coefficient (R)	P-value
Smoking	0.70	0.001*
Overweight	0.75	<0.001*
Income	−0.64	0.002*
Higher education	−0.80	<0.001*

*P-value <0.05.

## 4 Discussion

The difference in air quality among the 20 counties/cities was attributable to two reasons: natural factors and anthropogenic factors. In Fig. 1A, the green predominant area and the red predominant area was almost separated by the Central Mountain Range, which is an important geographic boundary of climate because the direction of the prevailing wind and the path of the weather system had significant intersection angle, caused the different regional climate in the same season [26], also a seasonal difference on the concentration of PM_2.5_ and PM10 was observed in a 9 years study, the highest mean PM_2.5_ particle concentration was detected during spring at the Banqiao (in New Taipei City), Hualien, and Taitung air quality monitoring stations [27], which may be exacerbated by the air pollution from China via northeast monsoon [28], and there was evidence showing that in spring, sulfate aerosols from remote sources were predominant in another study [29].

The major causes of air pollution in Taiwan were industrial exhaust (27.5%) and automobile emission (27.5%) according to the news of EPA, and the corresponding policies including the cap-and-trade program [30], allowance of replacing the old vehicle with new or electric one has been implemented [31]. Although some economic considerations blocking the enforcement was another obstacle that needed to be overcome [32], the reduction trend from 2005 to 2015, despite the vehicle numbers and energy consumption, industrial output, was similar to those of developed countries [33], implying that the air pollution policy was effective.

The incompatible result of AQI and air pollution caused death should be owing to the many confounding factors which played important parts in “air pollution caused death”, including smoking, being overweight, and higher education. A previous study in Taiwan found that not only fine particulate air pollution level, but also household income, physician density, high school graduate rate, smoking rate, and blue-collar worker percentage had a positive or negative effect on adult life expectancy [34], we needed a novel method to estimate the real air pollution caused death in Taiwan, the weight of each related disease must be adjusted based on further study to match the condition of Taiwan. Furthermore, this result also reminded us that we need to “treat underlying diseases” of “air pollution-caused death” if we want to reduce the mortality rate.

For the higher smoking rate in Taitung, although one of the most effective ways to reduce tobacco consumption was increasing tobacco taxes [[35], [36], [37], [38]], strangely, a 6-year-study in Taiwan revealed that there was no significantly quitting or reducing consumption of tobacco in most smokers because of non-tax-induced price increases, and some of them would even switching to cheaper brands within the same tobacco company [39], so other strategies different from increasing price should be used, such as a mobile application which was developed by Taiwanese researchers, and made use of a combination of I-Change behavioral change model with health recommender system and computer-tailoring for smoking cessation [40]. Moreover, some factors correlated with the success rate of smoking cessation should be early detected, including nicotine dependence level (measured by the FTCD), exhaled air carbon monoxide concentration, and cigarettes smoked per day which was proven by a Taiwanese study [41].

Overweight and even obesity have become an unneglectable health burden of Taitung for more than ten years, Taitung had the top-ranked adult obesity rate (48.1%) in Taiwan in 2011, and for obesity prevention, a series of strategies followed by Ottawa Charters Guideline were instituted from 2011, then decreased obesity rate (46.9%) with increased exercise rate (67.7%) were found at the end of 2012 (" [42], but the prevalence of overweight in Taitung was still the highest in the data obtained in 2019, therefore, the policies for weight control in Taitung could not only follow up Taiwan's Obesity Prevention and Management Strategy which was published by Health Promotion Administration [43], they should be customized based on the characteristics of Taitung, including the higher percentage of the aboriginal population (up to 30%) [44], the low-er-income and education accessibility, also the poor public transport [45].

Taitung County had no university until National Taitung University upgraded from National Taitung Teachers’ College in 2003, which was under the policy of “One county, (at least) one university” by President Shui-Bian Chen [46], indicating that the resource of higher education was exile, even though few studies investigated this issues, but some researchers evaluated the percentage of aboriginal students receiving the college education, the highest was in Taipei City (24.55%), and the lowest was in Taitung County (9.25%) which is almost the one-fourth of the means, and the drop-out rate even higher than the graduated rate, especially in Taitung County [47], then may cause that indigenous graduates tend to have lower salary and lower skill requirement jobs [48], and that should be the predisposing factor of health problems.

**Fig. 3:**
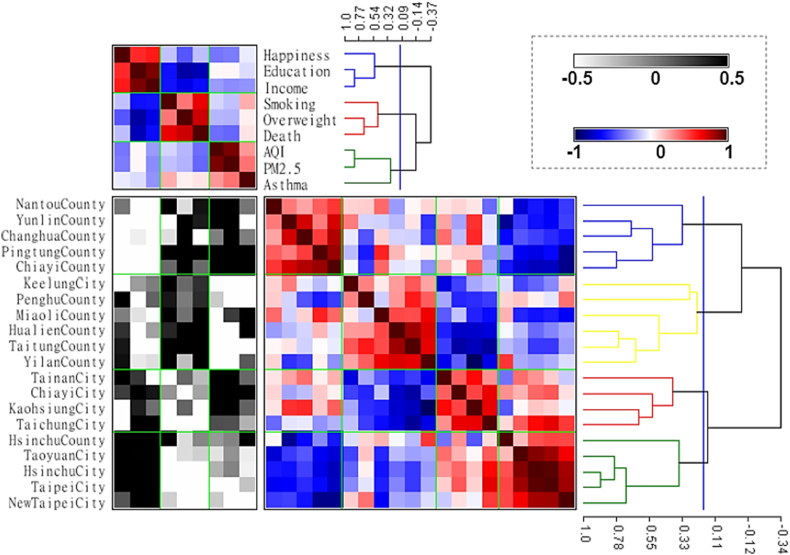
The combined matrices with clustering of health-related indicators and counties/cities. This figure consisted of three parts: the upper one was the proximity matrix of health-related indicators with the clustering tree, which was the same as Fig. 2. The left lower one was the raw data matrix, its columns were health-related indicators, and its rows were 20 counties/cities of Taiwan, all the data had been standardized, and the values lower than −0.5 were painted with the same color of −0.5, also the values upper than 0.5 were painted with the same color of 0.5 to demonstrate more clearly. The right lower one was the proximity matrix of 20 counties/cities of Taiwan with the clustering tree, and four groups were marked with different colors which would be used in Fig. 5 (blue, yellow, red, green).

The rural-urban disparity was also demonstrated in Fig. 3, Fig. 5, Group 1 and 2 contained all counties except Hsinchu County, and Groups 3 and 4 contained all cities except Keelung City. Cities had a higher level of “positive indicators” and a lower level of “negative indicators”, and then counties had a lower level of “positive indicators” and a higher level of “negative indicators” overall.

**Fig. 4:**
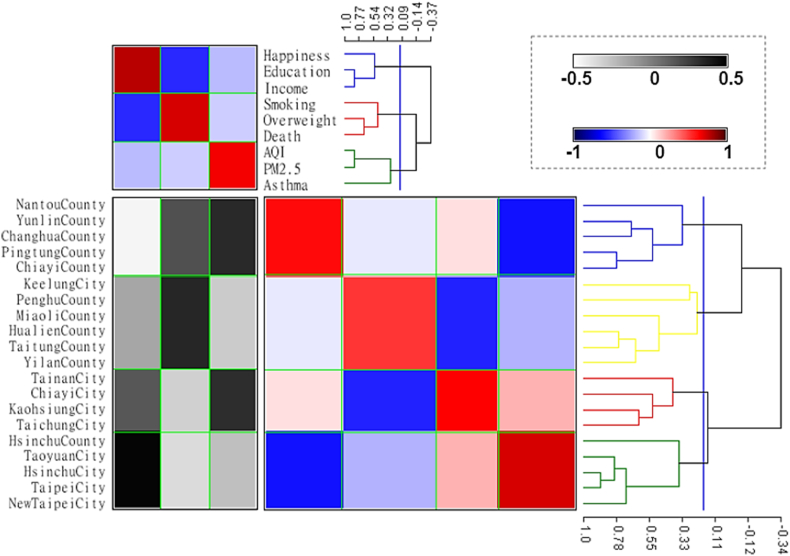
The combined matrices with clustering of health-related indicators and counties/cities, with proximity matrices partition and sufficient. Fig. 4 was the same matrix as Fig. 3 but underwent partition and sufficient with the means of each square to demonstrate the relationships between each group or cluster.

**Fig. 5:**
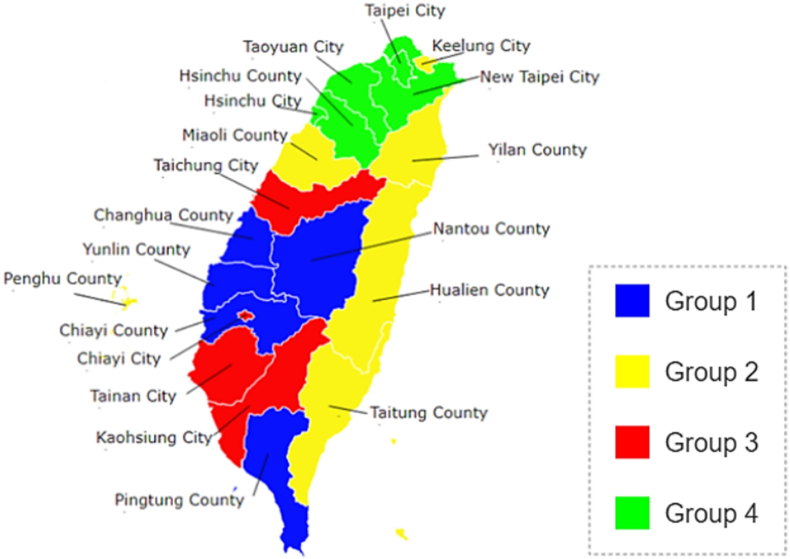
The distribution of the four groups of counties/cities on the map of Taiwan. Groups 1 & 3 could correspond to the yellow to red area (moderate to poor air quality) of the AQI visual map, while Groups 2 & 4 correspond to the area with good air quality (green of AQI map).

We found that Group 3 is special, it contained Tainan City, Kaohsiung City, and Taichung City, which were separated into three cities and three counties (Tainan City and County, Kaohsiung City and County, Taichung City and County) before “City-county Consolidation＂ on 2010/12/25 [49], so they had mixed characteristics of city and county, therefore, Group 3 had a moderately positive correlation to Group 4 (cities in the northern region) and mildly to moderately positive correlation to Group 1 (counties in the western region).

There are some limitations in this study, the major one is that we only evaluated the data in one year, and the time effect should also be considered. Perhaps a multi-year analysis could help us see more trends and changes in the relationship between air pollution and health problems. We chose 2019 to do this study because the medical resources and economy were relatively sufficient and stable compared to the years after the COVID-19 outbreak, perhaps the rural-urban disparity would be enlarged and the effect of air pollution on health would be decreased due to the new factor of a pandemic, which needs further studies to prove this hypothesis.

Moreover, some studies involved all the major air pollutants in their model, such as Lee et al.’s study presented on J Allergy Clin Immunol. in 2019, using a formula to combine these factors into a new value, then using this value to build their model ([50]. We did not involve all the air pollutants in our study because there’s no previous Taiwanese study that built the multivariable distributed lag nonlinear model for pollutants. Perhaps we can conduct another study by using the data from Taiwan to investigate whether our data can fit this model and whether the same effect also exists in Taiwan or not.

The reason why we involved PM_2.5_ in our generalized association plots is that a previous study showed PM_2.5_ is the most frequent primary air pollutant in Taiwan ([51], and our result also demonstrated that the Pearson’s correlation coefficient was more than 0.77 between AQI and PM_2.5_. Moreover, AQI is easy to understand and has been used in many studies to evaluate the relationship between health problems [52], indicating that AQI is representative enough for air quality.

In conclusion, this study initiated from the relationship between air quality and health problems in Taitung, and due to the incompatible result of AQI and air pollution caused death, we found that many confounding factors affected the relationship and may mask the protective effect of good air quality, then the rural-urban disparity was revealed by using some bio-psycho-social indicators via generalized association plots.

## Declarations

### Author contribution statement

Ching-Hsiang Yu: Performed the experiments; Analyzed and interpreted the data; Wrote the paper.

Shun-Chuan Chang: Analyzed and interpreted the data; Contributed reagents, materials, analysis tools or data; Wrote the paper.

En-Chih Liao: Conceived and designed the experiments; Wrote the paper.

### Funding statement

Professor En-Chih Liao was supported by 10.13039/501100014804Mackay Medical College [MMC-RD-110-1E-P004 & MMC-RD-111-1B-P018].

### Data availability statement

Data will be made available on request.

### Declaration of interest’s statement

The authors declare no competing interests.

### Ethics approval and consent to participate

Not applicable.

### Additional information

No additional information is available for this paper.

### Availability of data and materials

The data of AQI and PM_2.5_ were downloaded from Taiwan Air Quality Monitoring Network. https://airtw.epa.gov.tw/CHT/Query/His_Data.aspx.

The demographic data including income, smoking rate, and prevalence of overweight, causes of death were obtained from the Department of Statistics, Ministry of Health and Welfare. https://dep.mohw.gov.tw/DOS/lp-5069-113-xCat-y108.html.

The data on asthma attack incidence rate was retrieved from the Quality Performance of National Health Insurance Disclosure Website.

https://www.nhi.gov.tw/mqinfo/Map_1.aspx?Type=Asthma&DAID=1740&List=4.

The data on the happiness index of 2019 was gotten from Economy Daily News (Taiwan).

https://money.udn.com/ACT/2019/happy/chart/2019.pdf.

The percentage of residents who received higher education was downloaded from the Department of Household Registration, Ministry of the Interior.

https://www.ris.gov.tw/app/portal/346.

All the datasets in this study are available as references in the section of “Materials and Methods” and are also available from the corresponding author upon reasonable request.
